# Italian Version of the mHealth App Usability Questionnaire (Ita-MAUQ): Translation and Validation Study in People With Multiple Sclerosis

**DOI:** 10.2196/58079

**Published:** 2024-09-30

**Authors:** Jessica Podda, Erica Grange, Alessia Susini, Andrea Tacchino, Federica Di Antonio, Ludovico Pedullà, Giampaolo Brichetto, Michela Ponzio

**Affiliations:** 1 Scientific Research Area, Italian Multiple Sclerosis Foundation, Genoa, Italy; 2 Rehabilitation Service of Genoa, Italian Multiple Sclerosis Society, Genoa, Italy

**Keywords:** mHealth, multiple sclerosis, cognitive assessment, questionnaire validation, usability, mHealth app, mHealth application, validation study, MAUQ, app usability, telemedicine, disability, usability questionnaire, mobile health

## Abstract

**Background:**

Telemedicine and mobile health (mHealth) apps have emerged as powerful tools in health care, offering convenient access to services and empowering participants in managing their health. Among populations with chronic and progressive disease such as multiple sclerosis (MS), mHealth apps hold promise for enhancing self-management and care. To be used in clinical practice, the validity and usability of mHealth tools should be tested. The most commonly used method for assessing the usability of electronic technologies are questionnaires.

**Objective:**

This study aimed to translate and validate the English version of the mHealth App Usability Questionnaire into Italian (ita-MAUQ) in a sample of people with MS.

**Methods:**

The 18-item mHealth App Usability Questionnaire was forward- and back-translated from English into Italian by an expert panel, following scientific guidelines for translation and cross-cultural adaptation. The ita-MAUQ (patient version for stand-alone apps) comprises 3 subscales, which are ease of use, interface and satisfaction, and usefulness. After interacting with DIGICOG-MS (Digital Assessment of Cognitive Impairment in Multiple Sclerosis), a novel mHealth app for cognitive self-assessment in MS, people completed the ita-MAUQ and the System Usability Scale, included to test construct validity of the translated questionnaire. Confirmatory factor analysis, internal consistency, test-retest reliability, and construct validity were assessed. Known-groups validity was examined based on disability levels as indicated by the Expanded Disability Status Scale (EDSS) score and gender.

**Results:**

In total, 116 people with MS (female n=74; mean age 47.2, SD 14 years; mean EDSS 3.32, SD 1.72) were enrolled. The ita-MAUQ demonstrated acceptable model fit, good internal consistency (Cronbach *α*=0.92), and moderate test-retest reliability (intraclass coefficient correlation 0.84). Spearman coefficients revealed significant correlations between the ita-MAUQ total score; the ease of use (5 items), interface and satisfaction (7 items), and usefulness subscales; and the System Usability Scale (all *P* values <.05). Known-group analysis found no difference between people with MS with mild and moderate EDSS (all *P* values >.05), suggesting that ambulation ability, mainly detected by the EDSS, did not affect the ita-MAUQ scores. Interestingly, a statistical difference between female and male participants concerning the ease of use ita-MAUQ subscale was found (*P*=.02).

**Conclusions:**

The ita-MAUQ demonstrated high reliability and validity and it might be used to evaluate the usability, utility, and acceptability of mHealth apps in people with MS.

## Introduction

Telemedicine has enabled convenient and effective visits; reduced unnecessary testing and referrals; maintained good perception of care; reduced travel costs and caregiver burden; and, not least, helped health care providers to manage an ever-increasing volume of information and relationships [1,2]. Growing use of smartphones and tablets has made mobile health (mHealth) apps promising tools for empowering and engaging people in the self-management of their own health [3]. mHealth tools create opportunities to deliver new forms of health care and to expand services without the need to increase the existing workforce. For example, medical apps can be used within various domains such as wellness management, behavior change, health data collection, disease management, self-diagnosis, and rehabilitation as well as act as an electronic patient portal and medication reminder [4,5], leading to greater time spent at home and fewer medical visits at the center [6]. Furthermore, they represent useful solutions for participants with chronic and progressive diseases, such as multiple sclerosis (MS), that require continuous assistance and care.

mHealth apps promise to offer alternative methods for enhanced real-time data capture to screen for, monitor, and treat the heterogeneous symptoms in MS, thus favoring a substantial transformation in traditional paradigms of medicine [7-13]. However, as the number of mHealth apps increases, the demand for scientific evaluation of these solutions is strongly recommended as well [14]. Despite the growing popularity of mHealth apps, the amount of usability reports does not correlate with the number of published digital health implementation studies [15]. For instance, while Salimzadeh and colleagues [3] found 104 MS-related apps in iTunes (Apple Inc) and Google Play (Google LLC), they noted that there was no corresponding evidence regarding the usability and utility of these solutions in people with MS. To be used in clinical practice, the validity and usability of mHealth tools should be tested. mHealth apps must be designed to ensure good usability, and they must be easy to use and able to reach their goals efficiently. As indicated by Wilson and Lankton [16], perceived ease of use and usefulness affect people’s intention to adopt mHealth devices. Generally, a mobile app is considered to have good usability when (1) it is efficient, (2) users have a positive opinion about the app, (3) it is easy to learn, (4) it is easy to remember even after users have not used it for a while, and (5) it has a low error rate [17].

The mHealth apps can be grouped according to the nature of the interaction between patients and health care providers in the app: interactive and stand-alone mHealth apps. In interactive mHealth apps, users can send and receive information from their health care providers or patients via the app in a synchronous or asynchronous modality. In stand-alone mHealth apps, users enter, collect, or store health information about themselves or other people, which are not directly sent to the user’s health care providers [18].

The most commonly used method for assessing the usability of electronic technologies are questionnaires. General and technology-independent questionnaires such as the System Usability Scale (SUS) [19] and the Post-Study System Usability Questionnaire [20] are usually used in usability studies of mHealth apps [15]. However, these questionnaires were created for general software systems and cannot reliably identify mHealth specific problems that may arise, for example, in health self-management or accessing health care services.

In this context, a new specific usability scale for evaluating the validity of mHealth apps was developed, the mHealth App Usability Questionnaire (MAUQ) [18]. The English version of MAUQ has been translated to various languages such as Malay [21], Chinese [22], Spanish [23], German [24] and French [25]. However, no literature was found reporting a translated version of the questionnaire in Italian, although it was used in 1 study on app usability [26]. Thus, this study aimed to translate and validate the English version of the mHealth App Usability Questionnaire into Italian (ita-MAUQ) in a sample of people with MS.

We specifically hypothesized that the ita-MAUQ would retain acceptable model fit in confirmatory factor analysis, acceptable levels of internal consistency, and test-retest reliability. We also hypothesized an acceptable construct validity, defined based on relations between the ita-MAUQ and another standardized usability scale, and differences in known-groups.

## Methods

### mHealth App Usability Questionnaire

The MAUQ was first developed by Zhou and colleagues [18]. MAUQ is designed for different users (patients or health care providers) and different interaction modes (interactive or stand-alone). For study purposes, the patient version for stand-alone mHealth apps was used. It consists of 18 items divided into 3 subscales: ease of use (5 items; MAUQ_E), interface and satisfaction (7 items; MAUQ_I), and usefulness (6 items; MAUQ_U). The overall Cronbach α coefficients of the original questionnaire were 0.85 for MAUQ_E, 0.90 for MAUQ_I, and 0.72 for MAUQ_U, which indicated strong internal consistency of the questionnaire [18]. The questionnaire uses a 7-point Likert scoring system: 1 (strongly disagree), 2 (disagree), 3 (somewhat disagree), 4 (neither agree nor disagree), 5 (somewhat agree), 6 (agree), and 7 (strongly agree). The authors point out that there are no licensing fees for using the questionnaire, and it is not necessary to request permission before using it. The questionnaire is freely accessible on the website.

### MAUQ Translation and Cross-Cultural Adaptation

The original MAUQ questionnaire for stand-alone mHealth apps (patient version) was translated into the Italian language using a guideline for the translation, adaptation, and validation of instruments or scales for cross-cultural health care research [27]. The questionnaire was first translated by native Italian speakers proficient in English. The forward-translated versions of the instrument were initially compared by a third independent translator regarding ambiguities and discrepancies of words, sentences, and meanings to generate a preliminary initial translated version of the questionnaire. The back translation was verified by translating the Italian version to English by 2 native English speaker translators with a high Italian proficiency. A multidisciplinary panel with 3 health care professionals with expertise in MS (an occupational therapist, a physiotherapist, and a psychologist), 2 researchers with expertise in scale validation, and 1 expert in app development compared the translated versions with the original questionnaire and made modifications to make the questionnaire more understandable to Italian people with MS. The final ita-MAUQ questionnaire can be viewed in Multimedia Appendix 1.

### mHealth App Used for Validation of the Ita-MAUQ

**Figure 1.**
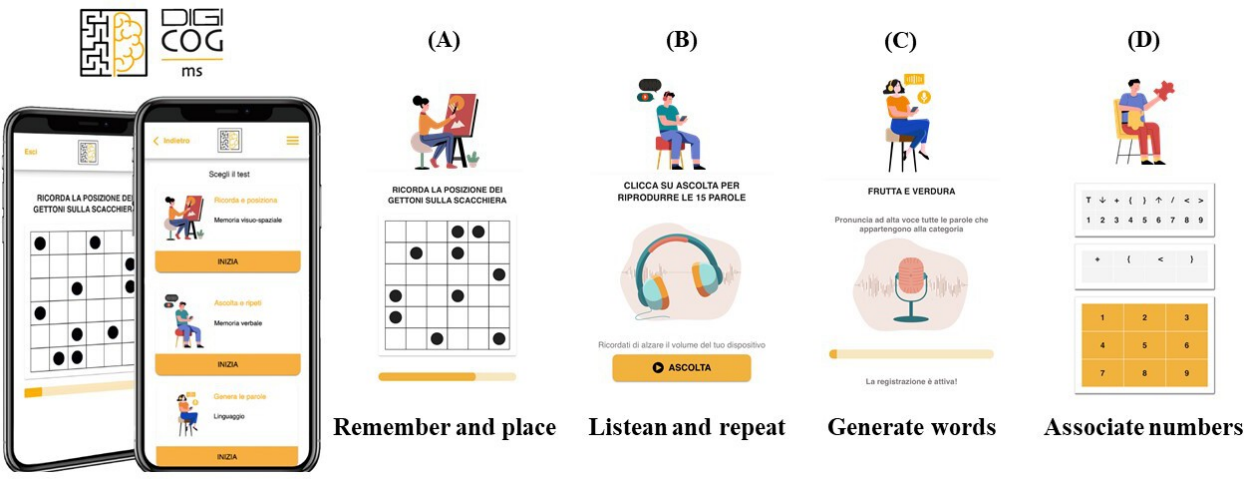
Overview of DIGICOG-MS, the mobile health for cognitive assessment of people with multiple sclerosis. The 4 digital tests implemented in DIGICOG-MS that measure visuospatial memory (**A**), verbal memory (**B**), semantic fluency (**C**), and information processing speed (**D**). DIGICOG-MS: Digital Assessment of Cognitive Impairment in Multiple Sclerosis.

In addition to motor and sensory difficulties, cognitive impairment, known as an invisible symptom, affects up to 65% of people with MS. Documented in all MS courses, with more severe deficits in progressive forms, both secondary progressive and primary progressive, compared to relapsing-remitting MS [28], cognitive impairment is recognized as one of the most disturbing disorders in MS, negatively affecting the quality of life and independence of people with MS. Attention, information processing speed, learning and memory, and executive functions seem to be the most commonly affected cognitive domains [29]. Consistent evidence indicates that cognitive functions in people with MS can be grouped into cognitive phenotypes, that is, subgroups of people with MS with a similar pattern of cognitive functioning [30-32]. The validation of the ita-MAUQ was conducted using DIGICOG-MS (Digital Assessment of Cognitive Impairment in Multiple Sclerosis), a smartphone- and tablet-based app for self-assessment of cognitive impairment in people with MS [33] (please see Figure 1 for an overview of the mHealth app). DIGICOG-MS (intellectual property of Italian Multiple Sclerosis Foundation; Italian Society of Authors and Publishers Registration ID: D000018162, 27-12-2022) includes 4 digital tests designed to evaluate the most affected cognitive domains in MS as visuospatial memory, verbal memory, semantic fluency, and information processing speed [34,35]:

- *Remember and place* assesses visuospatial episodic memory. A 36-square grid with 10 black checkers is displayed on the screen for 10 seconds. After the time elapses, the pattern disappears, and participants must reproduce it on a blank checkerboard. This replicates the 10/36 Spatial Recall Test [35], in which a 6 × 6 checkerboard with 10 pieces arranged in a particular pattern is shown to the participant for 10 seconds. Both tests (digital and traditional) include 3 consecutive trials, and the score consists of the total number of correct responses for the 3 trials.
- *Listen and repeat* was developed as an electronic version of the Rey Verbal Learning Test [36] that evaluates verbal memory. Participants listen to a prerecorded list of 15 common nouns and are asked to recall as many words as possible 5 times. Responses are recorded and then scored by the neuropsychologist. In the traditional test, words are read aloud to the participant who is asked to repeat as many words as possible in any order. All pronounced nouns in each of the 5 learning trials are transcribed by the neuropsychologist. For both versions of the test, the total score consists of the number of words recalled across the 5 trials.
- *Generate words* is a digital adaptation of the Word List Generation [35,37] and measures semantic verbal fluency. Participants generate a list of words*,* typically constrained by a specific semantic category, in 90 seconds. Recordings of pronounced words are processed by the neuropsychologist for scoring. In the traditional test, all words generated within the given semantic category are transcribed by the neuropsychologist. The total score is based on the number of correct words produced.

In a study by Podda and colleagues [33], correlation analysis was performed to determine the strength of the association between digital (ie, remember and place, listen and repeat, generate words, and associate numbers) and traditional (ie, 10/36 Spatial Recall Test, Rey Verbal Learning Test, Word List Generation, and Symbol Digit Modalities Test) tests. Overall, the findings revealed strong correlations between digital and traditional paper-based tests across all cognitive domains, with correlation coefficients (*r*) ranging from 0.58 to 0.78. Test-retest reliability was excellent for verbal memory and information processing speed (intraclass correlation coefficients [ICCs] ≥0.95) and good for visuospatial memory and semantic fluency (ICCs≥0.83).

### Study Participants

This study’s participants were people with MS enrolled by the Italian MS Foundation and followed as outpatients at the Italian Multiple Sclerosis Society Rehabilitation Service in Genoa (Italy). Eligibility criteria were to be aged 18 years or older; have a confirmed MS diagnosis following the McDonald criteria [38]; have any disease course (relapsing-remitting MS, secondary progressive MS, and primary progressive MS); have not relapsed in the last 3 months; have an Expanded Disability Status Scale (EDSS) score [39] ≤7.5; and have adequate visual, hearing, and motor capabilities to work on a tablet. Exclusion criteria were a Montreal Cognitive Assessment score <18, neurological and major psychiatric illness, past serious head trauma, and alcohol or drug abuse. Data were collected from January 2023 to November 2023.

### Study Procedure

Participants received a brief explanation of this study’s process and goal at the Rehabilitation Service of the Italian Multiple Sclerosis Society in Genoa (Italy). People with MS were invited to use DIGICOG-MS. Particularly, they were required first to perform the 4 digital cognitive tests and then to explore other functionalities of the app (ie, how to log in, insert, and modify personal information; check for historical results of a specific test; consult the app tutorial; and ask for support). While the digital cognitive assessment was supervised by a neuropsychologist, people with MS were invited to navigate through DIGICOG-MS autonomously, and no specific instructions on how to use other functionalities of the app were given. After finishing the tasks, people with MS completed the ita-MAUQ and SUS [19,40]. SUS was included to test construct validity of the translated questionnaire. The 10-item SUS evaluates users’ personal perceptions about how to use a given system or device, ranging from “strongly disagree” (1 point) to “strongly agree” (5 points). The SUS score is calculated by adding the individual scores and then multiplying that sum by 2.5. Thus, the SUS score ranges from zero (lowest usability) to 100 (highest usability), with a value of 68 considered above average. Although the SUS has already been translated and validated in many languages [41], individual problems that people may have when using mHealth apps are not specifically identified by the SUS.

### Statistical Analysis

Categorical data were summarized by numbers and percentages, while numerical data were indicated by the mean and SD. Degree of education was coded as less than or equal to 12 years (primary school), between 13 and 15 years (high school), and equal to or more than 16 years (university).

Since confirmatory factor analysis (CFA) has emerged as a pivotal technique in such contexts, offering a comprehensive method for comparing the hypothesized measurement model structure with the observed one [42], it was conducted on the 18-item ita-MAUQ using the original 3 higher-order factors structure (ie, MAUQ_E, MAUQ_I, and MAUQ_U) [18]. CFA was used instead of “discovering” or exploring potential relationships between variables, as in exploratory factor analysis, because it is designed to test a predefined model based on consistent theoretical expectations [43]. Goodness-of-fit was tested with the ratio between chi-square (*χ*^2^) and *df* (*χ*^2^/*df*; good if ≤3), root mean square error of approximation (good if ≤0.08) and comparative fit index >0.9 [44]. We reported these statistics using the Satorra-Bentler adjustment because the scale item distribution was nonnormal [45]; the covariance of error terms was considered to improve the model fit. In the data analysis, missing data were replaced with a value of 4.

The internal consistency of the ita-MAUQ was assessed by calculating Cronbach α coefficient and average interitem correlation. The statistically acceptable Cronbach α coefficient should be >0.7 [46], and average interitem correlations should be between 0.30 and 0.70 [47].

The ICC (2-way analysis of variance random effect model for agreement) was calculated to assess the test-retest reliability. A very small sample size is required for estimating the desired value of ICC (especially when a researcher aims to estimate a very high value of ICC). Using power analysis calculations to test reliability between 2 different observations as described by Bujang and Baharum [48], a minimum of 22 participants is needed to have an acceptable ICC value ≥0.5 (*α*=.05, power=80%, n=2). The ICC was calculated on subscales and total scores, which are expected to remain stable. An ICC value of 0.70 was recommended as a minimum standard for reliability [49].

Ceiling and floor effects were calculated for the overall ita-MAUQ and its subscales. The floor and ceiling effects were defined as the percentages of respondents who reported the lowest score and the highest score, respectively. Floor and ceiling effects were considered present if >15% of participants achieved either the lowest or highest scores in ita-MAUQ and its subscales [50].

To examine the construct validity, Spearman correlations coefficients (ρ), used for nonnormally distributed data, were calculated between the ita-MAUQ overall score and subscale scores, and the SUS. Spearman coefficients were considered low for ρ<0.30, moderate for ρ 0.30-0.59, and high for ρ≥0.60 [51].

Known-groups validity evaluates whether an instrument can discriminate between known groups of people with MS that are expected to score differently on the measure of interest (ie, EDSS and gender). Here, it was assessed by comparing, with the Mann-Whitney *U* test, the total score and subscale scores of participants’ groups with different levels of disability. Groups were defined using an EDSS cutoff value of 3.5, discriminating between people with MS with a mild and moderate disability: able (EDSS≤3.5) or unable (EDSS>3.5) to walk without aid or rest for more than 500 m. Furthermore, since previous research investigating gender differences in users’ acceptance for website usability highlights gender as a key variable in understanding usage behavior in information and communication technology [5,52,53], ita-MAUQ total score and subscale scores were also compared with groups divided by participants’ gender.

The *P* values <.05 were considered statistically significant. Data were analyzed using Stata (version 17; StataCorp).

### Ethical Considerations

This study was approved by Regional Ethics Committee of Azienda Ospedaliera “San Martino” of Genoa (Italy N. 240/2022DB id 12354) and conducted according to the Declaration of Helsinki [54]. Before entering this study, participants had to read, complete, and sign an informed consent. Data collected were stored in an anonymized format to properly protect the privacy and confidentiality of participants, ensuring that no participant can be identified from the data provided. Participants have been informed that data collection could be used only for research purposes. They did not receive any compensation for taking part in the study

## Results

In total, 116 people with MS (female: n=74; mean age 47.2, SD 14 years mean EDSS 3.32, SD 1.72) were enrolled (see Table 1).

Results from CFA of the 18 items-MAUQ order structure, defined by Zhou and colleagues [18], indicated acceptable fit for a 3D scale: *χ*^*2*^/*df*=2.1, root mean square error of approximation 0.068, and comparative fit index 0.92.

**Table 1.** Demographic and clinical sample characteristics (N=116).

Characteristics		Value
**Gender, n (%)**		
	Male	42 (36.2)
	Female	74 (63.8)
**Age (years)**		
	Mean (SD)	47.2 (14)
	Range	19‐70
Years of education, mean (SD)		13.3 (2.6)
**Education, n (%)**		
	Primary school	16 (13.8)
	High school	80 (69)
	University	20 (17.2)
**MS**^**a**^ **duration (years)**		
	Mean (SD)	11.2 (9.6)
	Range	0‐32
**MS course, n (%)**		
	RR^b^	91 (78.5)
	SP^c^	15 (12.9)
	PP^d^	10 (8.6)
**EDSS** ^**e**^		
	Mean (SD)	3.32 (1.72)
	Range	1‐7.5
**EDSS, n (%)**		
	Mild disability, score ≤3.5	67 (57.8)
	Moderate disability, score >3.5	49 (42.2)

a MS: multiple sclerosis.

b RR: relapsing-remitting multiple sclerosis.

c SP: secondary progressive multiple sclerosis.

d PP: primary progressive multiple sclerosis.

e EDSS: Expanded Disability Status Scale.

The internal consistency of the overall ita-MAUQ and each subscale was good. The Cronbach α for the ita-MAUQ was 0.92, and those for the 3 subscales were 0.78 (ita-MAUQ_E), 0.89 (ita-MAUQ_I), and 0.87 (ita-MAUQ_U). Similarly, the average interitem correlation was between 0.41 and 0.53. These results align with published satisfactory thresholds for scale reliability [23,24].

To assess the test-retest reliability, 25 participants were required to complete the ita-MAUQ, 2 weeks apart. ICC was 0.84 (95% CI 0.66‐0.92) for ita-MAUQ total score, 0.66 (95% CI 0.36‐0.84) for ita-MAUQ_E, 0.88 (95% CI 0.75‐0.94) for ita-MAUQ_I, and 0.67 (95% CI 0.38‐0.84) for ita-MAUQ_U, showing good or moderate temporal stability.

There were no floor or ceiling effects for the ita-MAUQ total score (0% scored 18 and 6.9% scored 126). The floor effect for ita-MAUQ_E and ita-MAUQ_I subscales was again null, while for ita-MAUQ_U, only 1 participant had the worst possible score of 6 (0.9%). The ceiling effect for ita-MAUQ subscales was 38.8%, 36.2%, and 9.5%, respectively. No other previous study reported the measure of ceiling and floor effects of this instrument [22-24], and therefore a comparison cannot be established.

Spearman coefficients revealed significant correlations between ita-MAUQ total score; ita-MAUQ_E, ita-MAUQ_I, ita-MAUQ_U subscales; and SUS (85.43, SD 14.3; all *P* values <.05). Table 2 shows the results of the construct validity analysis.

Known-group analysis found no difference between people with MS with mild and moderate EDSS (all *P* values >.05), suggesting that ambulation ability, mainly detected by the EDSS, did not impact the ita-MAUQ scores (Table 3). Interestingly, statistical differences between female and male participants concerning the ita-MAUQ_E was found (*P*=.02; Table 4).

**Table 2.** Spearman correlation (ρ) between the ita-MAUQ total^a^ score; ita-MAUQ_E^b^, ita-MAUQ_I^c^, and ita-MAUQ_U^d^ subscales; and SUS^e^ total score.

Variable		Value, mean (SD)	SUS	ita-MAUQ_E	ita-MAUQ_I	ita-MAUQ_U	ita-MAUQ_tot
**SUS**		85.43 (14.3)					
	*r*		—^f^				
	*P value*		—				
**ita-MAUQ_E**		31.7 (4.1)					
	*r*		0.54	—			
	*P* value		<.001	—			
**ita-MAUQ_I**		44.3 (6.3)					
	*r*		0.60	0.76	—		
	*P* value		<.001	<.001	—		
**ita-MAUQ_U**		31.8 (6.8)					
	*r*		0.26	0.38	0.48	—	
	*P* value		.005	<.001	<.001	—	
**ita-MAUQ_tot**		107.8 (14.5)					
	*r*		0.53	0.78	0.85	0.80	—
	*P* value		<.001	<.001	<.001	<.001	—

a ita-MAUQ_tot: Italian version of the mHealth App Usability Questionnaire.

b ita-MAUQ_E: ease of use subscale of the Italian version of the mHealth App Usability Questionnaire.

c ita-MAUQ_I: interface and satisfaction subscale of the Italian version of the mHealth App Usability Questionnaire.

d ita-MAUQ_U: usefulness subscale of the Italian version of the mHealth App Usability Questionnaire.

e SUS: System Usability Scale.

f Not applicable.

**Table 3.** Comparison of ita-MAUQ total^a^ and subscale scores between people with multiple sclerosis with different disability levels.

Variable	ita-MAUQ_E^b^, mean (SD)	ita-MAUQ_I^c^, mean (SD)	ita-MAUQ_U^d^, mean (SD)	ita-MAUQ_tot, mean (SD)
EDSS^e^≤3.5	31.9 (3.6)	44.1 (6.2)	31.0 (6.9)	107.0 (13.9)
EDSS>3.5	31.4 (4.7)	44.7 (6.7)	32.9 (6.6)	109.0 (15.5)
*P* value	.76	.28	.10	.24

a ita-MAUQ_tot: Italian version of the mHealth App Usability Questionnaire.

b ita-MAUQ_E: ease of use subscale of the Italian version of the mHealth App Usability Questionnaire.

c ita-MAUQ_I: interface and satisfaction subscale of the Italian version of the mHealth App Usability Questionnaire.

d ita-MAUQ_U: usefulness subscale of the Italian version of the mHealth App Usability Questionnaire.

e EDSS: Expanded Disability Status Scale.

**Table 4.** Comparison of ita-MAUQ total^a^ and subscale scores between people with multiple sclerosis by gender.

Variable	ita-MAUQ_E^b^ mean (SD)	ita-MAUQ_I^c^ mean (SD)	ita-MAUQ_U^d^ mean (SD)	ita-MAUQ_tot mean (SD)
Female	32.4 (3.6)	45.1 (6.0)	32.0 (7.2)	109.5 (14.3)
Male	30.5 (4.6)	43.1 (6.9)	31.4 (6.1)	104.9 (14.8)
*P* value	.02	.09	.51	.06

a ita-MAUQ_tot: Italian version of the mHealth App Usability Questionnaire.

b ita-MAUQ_E: ease of use subscale of the Italian version of the mHealth App Usability Questionnaire.

c ita-MAUQ_I: interface and satisfaction subscale of the Italian version of the mHealth App Usability Questionnaire.

d ita-MAUQ_U: usefulness subscale of the Italian version of the mHealth App Usability Questionnaire.

## Discussion

Digital solutions as mHealth apps promise to offer alternative methods for enhanced real-time data capture to screen for, monitor, and treat symptoms in MS. These solutions may fundamentally shift traditional paradigms of medicine [7-12]. To be used in clinical practice, the validity and usability of mHealth tools should be tested. Questionnaires are the well-known methods for usability testing, but developing a new one might require concerted effort by the members of a research team, extra cost, and a lot of time [55]. Thus, adaptation of established, appropriate, and available questionnaires with documented validity in other languages is recommended [55].

Thus, the aim of this study was to translate and validate the English version of the ita-MAUQ in a sample of people with MS. Overall, findings demonstrated that the novel translated questionnaire ita-MAUQ is a reliable and valid measurement tool to assess the usability of mHealth apps for people with MS. In this context, people with MS self-administered the ita-MAUQ after interacting with DIGICOG-MS, a novel mHealth app for cognitive self-assessment in MS.

Results indicated that the ita-MAUQ had good internal consistency and stability, as indicated by the Cronbach α coefficient of 0.92. This is in line with the original version of the MAUQ [18] and with other translations of the same questionnaire [23,24,56].

Worldwide, Spearman coefficients between ita-MAUQ total score, subscales, and SUS were statistically significant, proving good criterion and construct validity. However, correlation between the SUS and ita-MAUQ_U was found to be low (0.26). This was in line with the study by Zhou et al [18], in which correlation between MAUQ_U and the SUS was 0.383, reflecting that MAUQ_U is mainly about the usefulness of apps for health care, which is an aspect not covered by the SUS.

Compared to another previous study on MAUQ that did not perform test-retest [24], results revealed good or moderate temporal stability. Given that mHealth apps may allow continuous health care services over time, identifying valid methods to test whether a digital tool is reliable in different measurements is crucial.

The ceiling effect of the ita-MAUQ subscores could indicate that the proposed mHealth app was indeed easy to use for many people with MS. Even though the interaction with DIGICOG-MS was supervised by a neuropsychologist to provide adequate responses to any questions from participants, they were invited to navigate through the mHealth app autonomously, since no specific instructions on how to use other app functionalities were given. In this way, we interpreted this ceiling effect positively as a successful interaction.

In general, the known-groups validity of the instrument was shown by the comparison of ita-MAUQ total and subscale scores between people with MS with different disability levels. The results indicate no significant difference in people with MS with mild and moderate EDSS. Since EDSS mainly measured ambulation capacity, this suggests that such mHealth apps could be found usable for both people that need assistance during ambulation and those who are able to walk without any aid or support.

Concerning gender, earlier studies found that perceived ease of use and usefulness technology may differ by gender [5,52]. Our results are in line with a previous study that demonstrated that gender was associated with higher usability scores in female than male participants [57]. Interestingly, male and female people with MS had different scores in ita-MAUQ_E, suggesting that women are more likely than men to be influenced by effort expectancy and facilitating conditions [53]. These results can help developers to enhance the usability of their services for all users with different personal and clinical characteristics, since men and women still have different traits and societal roles, which may affect their perceptions and usage of technologies.

Our study has several limitations. First, as a single-center study, participants’ characteristics may limit the interpretation of our results. The study sample may be considered representative of those clinic-attending people with MS followed as outpatients in rehabilitation centers (ie, middle-age or older adults and with a longer disease duration) [58]. Thus, results may not generalize to other populations of participants with MS (eg, young and neo-diagnosed people). Second, in this validation study, we used DIGICOG-MS that was designed and developed to assess a specific symptom in MS, that is cognitive impairment. Given the high frequency of cognitive impairment in people with MS, it is reasonable to conclude that study participants who have experienced such a disturbing symptom tended to appreciate the mHealth app more compared to people with other neurological diseases. People with MS with cognitive impairment might find such digital tools particularly beneficial due to their potential to offer structured cognitive training and monitoring, which can enhance their daily functioning and quality of life [59]. Thus, it cannot be ruled out that the results of the ita-MAUQ validation would have been different with a clinical population with dissimilar characteristics.

Third, this study was conducted in a controlled clinical setting, allowing participants to familiarize themselves with both the novel technology and usability questionnaires’ items. While this approach may have some limitations in terms of generalizability to real-life scenarios and may influence participants’ engagement and perceived usability, having a facilitator available to assist, if needed, people with MS that could have problems with reading and interpreting the questionnaire items ensured they understand each question before responding. Furthermore, in this study we overlooked other key factors that may influence usability, such as assessing the importance of providing adequate training and continuous support to users or the role of previous experience with a similar technology; investigating how interface design that considering layout, navigation, and accessibility features makes the app user-friendly for participants with cognitive impairments; evaluating the concerns and preferences of users regarding data security and privacy, which are crucial for building trust and ensuring compliance with health regulations data security and privacy. Here, we did not collect additional feedback after the completion of both digital assessment and usability questionnaires from people with MS. Further study should include feedback sections where participants can indicate if they found any items difficult to understand, allowing for continuous improvement of the novel tools. Worldwide, incorporating these factors into future research can lead to the development of more effective, user-friendly, and impactful mHealth apps for people with MS.

In conclusion, the ita-MAUQ demonstrated high reliability and validity, and it might be used to evaluate the usability, utility, and acceptability of mHealth apps in people with MS. This finding is in line with previous validation of the MAUQ in different languages as Malay [21], Chinese [22], Spanish [23], German [24], and French [25], further confirming the cross-cultural validity, reliability, and adaptability of the MAUQ.

## Supplementary material

10.2196/58079 Multimedia Appendix 1 The Italian version of the mHealth App Usability Questionnaire (ita-MAUQ; for stand-alone mHealth apps used by patients).

## Abbreviations

CFA: confirmatory factor analysis
DIGICOG-MS: Digital Assessment of Cognitive Impairment in Multiple Sclerosis
EDSS: Expanded Disability Status Scale
ICC: intraclass correlation coefficient
ita-MAUQ: Italian version of the mHealth App Usability Questionnaire
ita-MAUQ_E: ease of use subscale of the Italian version of the mHealth App Usability Questionnaire
ita-MAUQ_I: interface and satisfaction subscale of the Italian version of the mHealth App Usability Questionnaire
ita-MAUQ_U: usefulness subscale of the Italian version of the mHealth App Usability Questionnaire
MAUQ: mHealth App Usability Questionnaire
MAUQ_E: ease of use subscale
MAUQ_I: interface and satisfaction subscale
MAUQ_U: usefulness subscale
mHealth: mobile health
MS: multiple sclerosis
SUS: System Usability Scale
