# Supplementary material for: Italian Version of the mHealth App Usability Questionnaire (Ita-MAUQ): Translation and Validation Study in People With Multiple Sclerosis
Source: JMIR Hum Factors. 2024 Sep 30;11:e58079. doi: 10.2196/58079 (PMC11457702; doi:10.2196/58079)
Supplement: Multimedia Appendix 1 [file humanfactors-v11-e58079-s001.docx]

**The Italian mHealth App Usability Questionnaire (ita-MAUQ)**

*Standalone mHealth Apps Used by Patients*

Il questionario ita-MAUQ è uno strumento progettato per valutare l'usabilità delle app per la salute (mHealth app).

La preghiamo di indicare il suo livello di accordo con ciascuna affermazione relativa alla sua esperienza con l’app che ha utilizzato, ponendo una “X” sotto l’opzione appropriata.

Se non può rispondere ad un’affermazione, la invitiamo a segnare “N/A” (non so, non valutabile).

|  | **N/A** | **totale disaccordo** | **disaccordo** | **parziale disaccordo** | **né in disaccordo né accordo** | **parziale accordo** | **accordo** | **totale accordo** |
| --- | --- | --- | --- | --- | --- | --- | --- | --- |
| ***Facilità d’uso*** |  |  |  |  |  |  |  |  |
| 1. L'app è stata facile da usare | ☐ | 1 | 2 | 3 | 4 | 5 | 6 | 7 |
| 2. È stato facile per me imparare ad usare l'app | ☐ | 1 | 2 | 3 | 4 | 5 | 6 | 7 |
| 3. La navigazione tra le schermate dell'app era coerente | ☐ | 1 | 2 | 3 | 4 | 5 | 6 | 7 |
| 4. L'interfaccia dell'app mi ha consentito di usare tutte le funzionalità offerte (es. inserire informazioni, rispondere a promemoria, prendere visione di informazioni) | ☐ | 1 | 2 | 3 | 4 | 5 | 6 | 7 |
| 5. Quando ho commesso un errore nell'uso dell'app, sono riuscita/o a correggerlo facilmente e rapidamente | ☐ | 1 | 2 | 3 | 4 | 5 | 6 | 7 |
| ***Interfaccia e soddisfazione*** |  | | | | | | | |
| 6. Mi piace l'interfaccia dell'app | ☐ | 1 | 2 | 3 | 4 | 5 | 6 | 7 |
| 7. Le informazioni presenti nell'app erano così ben organizzate da farmi trovare facilmente quello di cui avevo bisogno | ☐ | 1 | 2 | 3 | 4 | 5 | 6 | 7 |
| 8. L'app ha fornito informazioni adeguate a comprendere l'avanzamento delle mie azioni | ☐ | 1 | 2 | 3 | 4 | 5 | 6 | 7 |
| 9. Mi sento a mio agio ad usare l'app nei contesti sociali | ☐ | 1 | 2 | 3 | 4 | 5 | 6 | 7 |
| 10. La quantità di tempo dedicato all'uso dell'app è adeguata | ☐ | 1 | 2 | 3 | 4 | 5 | 6 | 7 |
| 11. Userei questa app in futuro | ☐ | 1 | 2 | 3 | 4 | 5 | 6 | 7 |
| 12. Globalmente, sono soddisfatta/o di questa app | ☐ | 1 | 2 | 3 | 4 | 5 | 6 | 7 |
| ***Utilità*** |  |  |  |  |  |  |  |  |
| 13. L'app sarebbe utile per la mia salute e il mio benessere | ☐ | 1 | 2 | 3 | 4 | 5 | 6 | 7 |
| 14. L'app ha migliorato l'accesso ai servizi di assistenza | ☐ | 1 | 2 | 3 | 4 | 5 | 6 | 7 |
| 15. L'app mi ha aiutato nel gestire efficacemente la mia salute | ☐ | 1 | 2 | 3 | 4 | 5 | 6 | 7 |
| 16. L'app presenta tutte le funzionalità e possibilità che mi aspettavo | ☐ | 1 | 2 | 3 | 4 | 5 | 6 | 7 |
| 17. Sono riuscita/o ad usare l'app anche quando la connessione internet era scarsa o non disponibile | ☐ | 1 | 2 | 3 | 4 | 5 | 6 | 7 |
| 18. Questa app ha fornito un modo affidabile per accedere ai servizi di assistenza, come fruizione di materiale informativo, monitoraggio delle attività ed auto-valutazione | ☐ | 1 | 2 | 3 | 4 | 5 | 6 | 7 |

La traduzione e validazione italiana del questionario è stata realizzata da *Podda J, Grange E, Susini A, Tacchino A, Di Antonio F, Pedullà L, Brichetto G, Ponzio M. Italian Version of the mHealth App Usability Questionnaire (Ita-MAUQ): Translation and Validation Study in People With Multiple Sclerosis. JMIR Hum Factors 2024;11: e58079.* Si prega di citare gli autori in caso di un utilizzo del questionario.
